# Retrosternal Multinodular Goiter With Extensive Mediastinal Extension Requiring Combined Cervical Thyroidectomy and Median Sternotomy: A Case Report

**DOI:** 10.7759/cureus.108106

**Published:** 2026-05-01

**Authors:** Jonathan A Charles, Stairis P Vinoj, Steles P Vinoj, Chirag Gaba, Akash Rahangan, Dheenu S

**Affiliations:** 1 Department of Medicine and Surgery, SRM Medical College Hospital and Research Centre, Chengalpattu, IND; 2 Department of Pharmacology, SRM Medical College Hospital and Research Centre, Chengalpattu, IND; 3 Department of General Surgery, SRM Medical College Hospital and Research Centre, Chengalpattu, IND

**Keywords:** case reports, mediastinum, multinodular goiter, retrosternal goiter, sternotomy, substernal goiter, thyroidectomy, thyroid gland, thyroid goiter, thyroid neoplasms

## Abstract

Retrosternal goiter is a distinct clinical entity, characterized by extension of thyroid tissue into the mediastinum, often increasing surgical complexity and necessitating individualized operative planning. We report a 55-year-old female patient presenting with a gradually enlarging anterior neck swelling without compressive or thyrotoxic symptoms. Clinical examination suggested mediastinal extension, and imaging with contrast-enhanced computed tomography confirmed a large multinodular goiter extending retrosternally up to the level of the D7 vertebra, abutting major mediastinal vessels without encasement. Fine-needle aspiration cytology was benign (Bethesda Category II). In view of the significant intrathoracic extension and proximity to vital structures, the patient underwent total thyroidectomy with a combined cervical approach and median sternotomy. Histopathological examination confirmed multinodular goiter without evidence of malignancy. The postoperative course was uneventful, and the patient remained asymptomatic on follow-up. This case highlights the importance of preoperative imaging in defining mediastinal extent and guiding surgical approach, emphasizing that extensive retrosternal extension and vascular proximity may necessitate a combined cervicothoracic approach to ensure safe and complete excision with minimal complications.

## Introduction

Retrosternal goiter, also termed substernal or intrathoracic goiter, is defined as an enlargement of the thyroid gland with extension below the thoracic inlet into the mediastinum [1]. From a general surgical perspective, it represents a distinct clinical entity because mediastinal extension alters anatomical relationships, operative exposure, and perioperative risk. Classical surgical descriptions emphasize that the inferior border of the gland is not clinically palpable and that a variable proportion of the thyroid mass lies within the superior mediastinum [2].

The reported incidence of retrosternal extension among patients undergoing thyroidectomy ranges from 5% to 20%, depending on the definition employed and the population studied [3]. In the majority of cases, the pathology represents secondary descent of a long-standing multinodular goiter rather than a primary ectopic mediastinal thyroid. Institutional analyses have demonstrated that these lesions typically retain cervical vascular supply, an observation that is critical in operative planning and vascular control [4]. Understanding this anatomical continuity allows the surgeon to anticipate that most cases can be approached cervically, while recognizing situations that may necessitate an extracervical extension.

Clinically, retrosternal goiter may remain asymptomatic for years. Progressive enlargement can eventually produce compressive symptoms such as dyspnea, orthopnea, dysphagia, or voice change due to tracheal or recurrent laryngeal nerve compression [5]. Examination findings suggestive of mediastinal extension include inability to palpate the lower pole of the thyroid, tracheal deviation, and features of thoracic inlet obstruction. Although the predominant pathology is benign multinodular goiter, the incidence of malignancy is comparable to cervical nodular disease and must be excluded through systematic evaluation [6].

Most patients are euthyroid at presentation, though hypo- or hyperthyroidism may coexist depending on underlying pathology [7]. Current guideline-based recommendations advocate comprehensive assessment including thyroid function testing, ultrasonography, and fine-needle aspiration cytology where indicated, ensuring appropriate risk stratification prior to surgical intervention [8]. Such evaluation is essential in determining the extent of surgery and anticipating postoperative management.

Cross-sectional imaging assumes central importance in the surgical workup. While ultrasonography delineates cervical disease, contrast-enhanced computed tomography of the neck and thorax is considered the investigation of choice to define the extent of mediastinal involvement, degree of tracheal compression, and relationship to major vascular structures [9]. Accurate preoperative imaging assists the surgeon in predicting the need for sternotomy, particularly when the goiter extends beyond the aortic arch or occupies the posterior mediastinum.

Despite improvements in iodine supplementation programs globally, goiter remains prevalent in certain geographic regions, and long-standing multinodular enlargement continues to be encountered in surgical practice [10]. Progressive inferior migration of an untreated goiter can culminate in intrathoracic extension, reinforcing the importance of timely evaluation and definitive management.

Surgical excision remains the definitive treatment for retrosternal goiter. Although the majority of cases can be managed via a standard cervical approach, a minority require median sternotomy or combined cervicothoracic access to ensure safe dissection and complete excision, particularly when there is significant mediastinal extension or vascular proximity [1,5]. Meticulous preoperative planning, multidisciplinary coordination, and adherence to sound surgical principles are essential to minimize complications such as recurrent laryngeal nerve injury, hypocalcemia, and major vascular injury [5].

The present case highlights a large multinodular goiter with retrosternal extension necessitating combined cervical and sternotomy approach. Reporting such cases contributes to surgical literature by reinforcing clinical indicators of mediastinal involvement, imaging-guided operative planning, and structured decision-making in complex thyroid surgery.

## Case presentation

A 55-year-old female patient, a homemaker by occupation and resident of a hill region, presented to the General Surgery outpatient department with a history of swelling in the anterior aspect of the neck for six months. The swelling was initially small and insidious in onset. Over the subsequent three months, it gradually increased in size to reach its present dimensions. There was no associated pain, redness, or local inflammatory features. The patient denied symptoms suggestive of pressure effects such as dyspnea, orthopnea, dysphagia, stridor, or change in voice. There was no history suggestive of hyperthyroidism or hypothyroidism. She did not report weight loss, loss of appetite, or features suggestive of malignancy.

Her past medical history was significant for type 2 diabetes mellitus and systemic hypertension, both on regular medical therapy. There was no prior history of thyroid disease, neck irradiation, or previous neck surgery. Family and menstrual histories were unremarkable.

Clinical examination

On general examination, the patient was well-built and well-nourished. There was no pallor, icterus, cyanosis, clubbing, lymphadenopathy, or pedal edema. Vital parameters were stable with pulse rate 78/min, blood pressure 110/70 mmHg, and oxygen saturation 99% on room air.

Local examination of the neck revealed a visible swelling measuring approximately 10 × 8 × 3 cm in the anterior neck region corresponding to the thyroid area. The superior border extended up to the level of the thyroid cartilage. The inferior border was not visible on inspection. Laterally, the swelling extended up to 0.5 cm medial to the anterior border of the sternocleidomastoid muscle on the right side, while the left lateral limit was not clearly appreciable. The swelling was irregular in contour with a bosselated surface. Overlying skin was normal, without scars, sinus, or dilated veins. No visible pulsations were noted.

On palpation, the swelling was firm in consistency, non-tender, and moved with deglutition. The lower border was not palpable. Multiple nodules were appreciated in both lobes of the thyroid gland, with the largest nodule measuring approximately 2 × 3 cm in the upper pole of the left lobe. The swelling was deep to the deep cervical fascia. Trachea was clinically central. There was no palpable thrill or bruit. Cervical lymph nodes were not enlarged. Pemberton’s sign was negative.

Percussion over the sternum revealed dullness, raising suspicion of retrosternal extension. Auscultation did not reveal any bruit. Systemic examination of cardiovascular, respiratory, abdominal, and neurological systems was within normal limits.

Investigations

Routine laboratory investigations were within normal limits. Thyroid function tests were within reference range, indicating a euthyroid state. Electrocardiogram was normal.

Chest X-ray showed mediastinal widening. Fine-needle aspiration cytology (FNAC) revealed benign thyroid follicular cells arranged in clusters with abundant colloid background, consistent with Bethesda Category II [11]. Histopathological correlation was also performed.

Contrast-enhanced computed tomography (CECT) of the neck and thorax demonstrated diffuse enlargement of the thyroid gland with retrosternal extension. The gland measured approximately 10.2 × 4.8 × 8.0 cm, with inferior extension of approximately 4 cm below the thoracic inlet, reaching up to the level of the D7 vertebra. The mass was seen abutting the sternum anteriorly and the aorta posteriorly, without evidence of vascular encasement or tracheal compression. CECT images could not be included due to suboptimal image quality; however, the radiological findings were clearly documented and guided surgical decision-making.

Operative management

In view of the significant retrosternal extension and proximity to mediastinal structures, cardiothoracic surgical consultation was obtained. The patient was planned for total thyroidectomy with median sternotomy for safe excision of the retrosternal component.

A cervical approach was considered initially; however, based on preoperative imaging demonstrating deep mediastinal extension and vascular proximity, a primary combined cervicosternotomy approach was planned to avoid intraoperative complications and ensure complete excision. Although most retrosternal goiters can be managed via a cervical approach, preoperative imaging in our case demonstrated deep mediastinal extension up to the D7 vertebral level, with posterior relation to the aorta and indentation over the innominate artery. The configuration suggested an “iceberg” pattern, with a significant intrathoracic component, making cervical delivery potentially unsafe. In view of these anatomical considerations and proximity to major vascular structures, a primary combined cervicosternotomy approach was planned.

**Figure 1:**
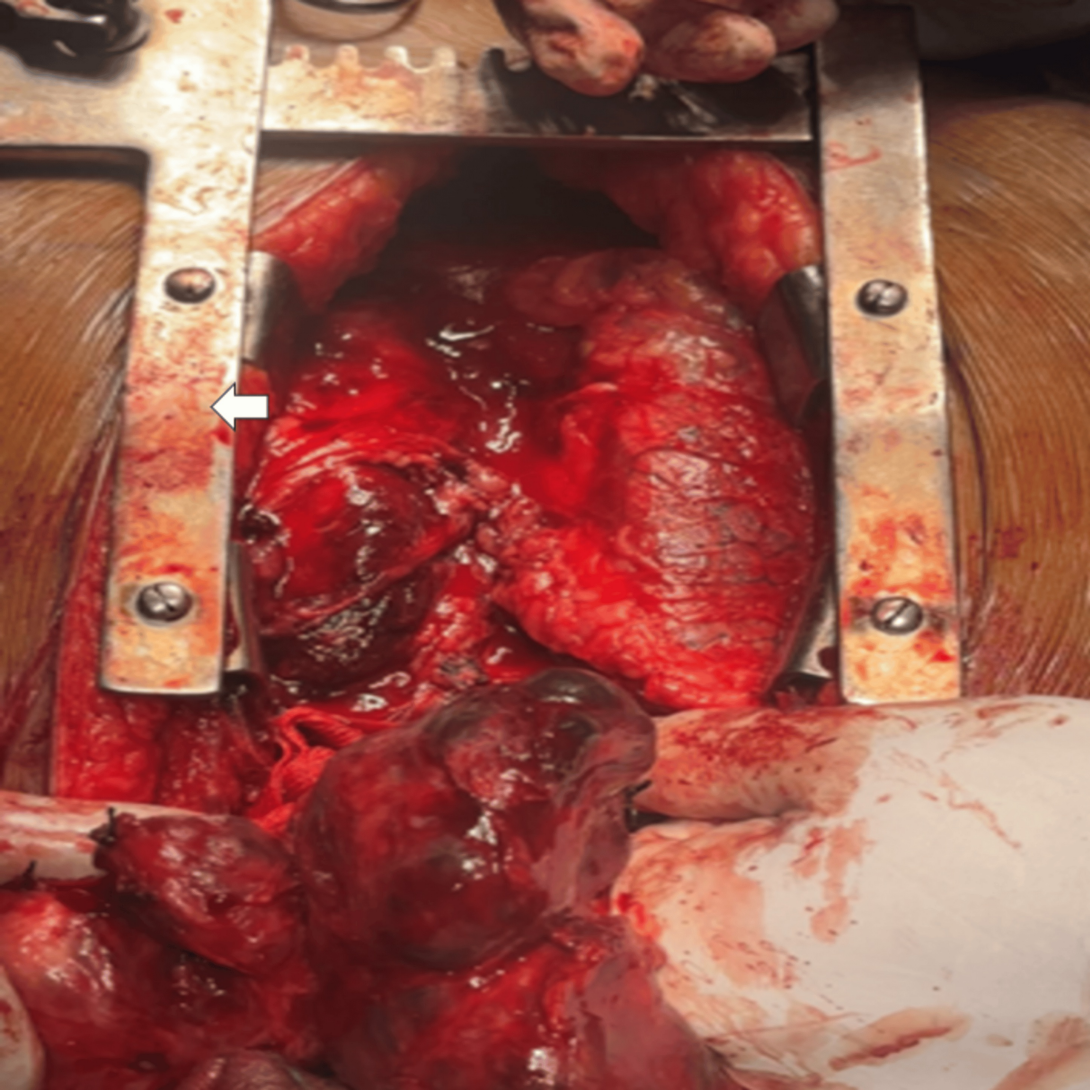
Intraoperative image showing median sternotomy with exposure of the mediastinum The sternum is seen anteriorly (arrowhead), with underlying mediastinal structures visible following retraction. The operative field demonstrates access to the retrosternal component of the multinodular goiter. The superior aspect is oriented towards the top of the image and the inferior aspect towards the bottom.

Under general anesthesia, a combined cervicosternotomy approach was performed. A transverse cervical incision was made, and subplatysmal flaps were raised. The strap muscles were separated in the midline to expose the thyroid gland. Superior pole vessels were ligated and divided, followed by capsular dissection with careful preservation of the recurrent laryngeal nerves and parathyroid glands. In view of the significant retrosternal extension, median sternotomy was performed to access the mediastinal component (Figure 1).

**Figure 2:**
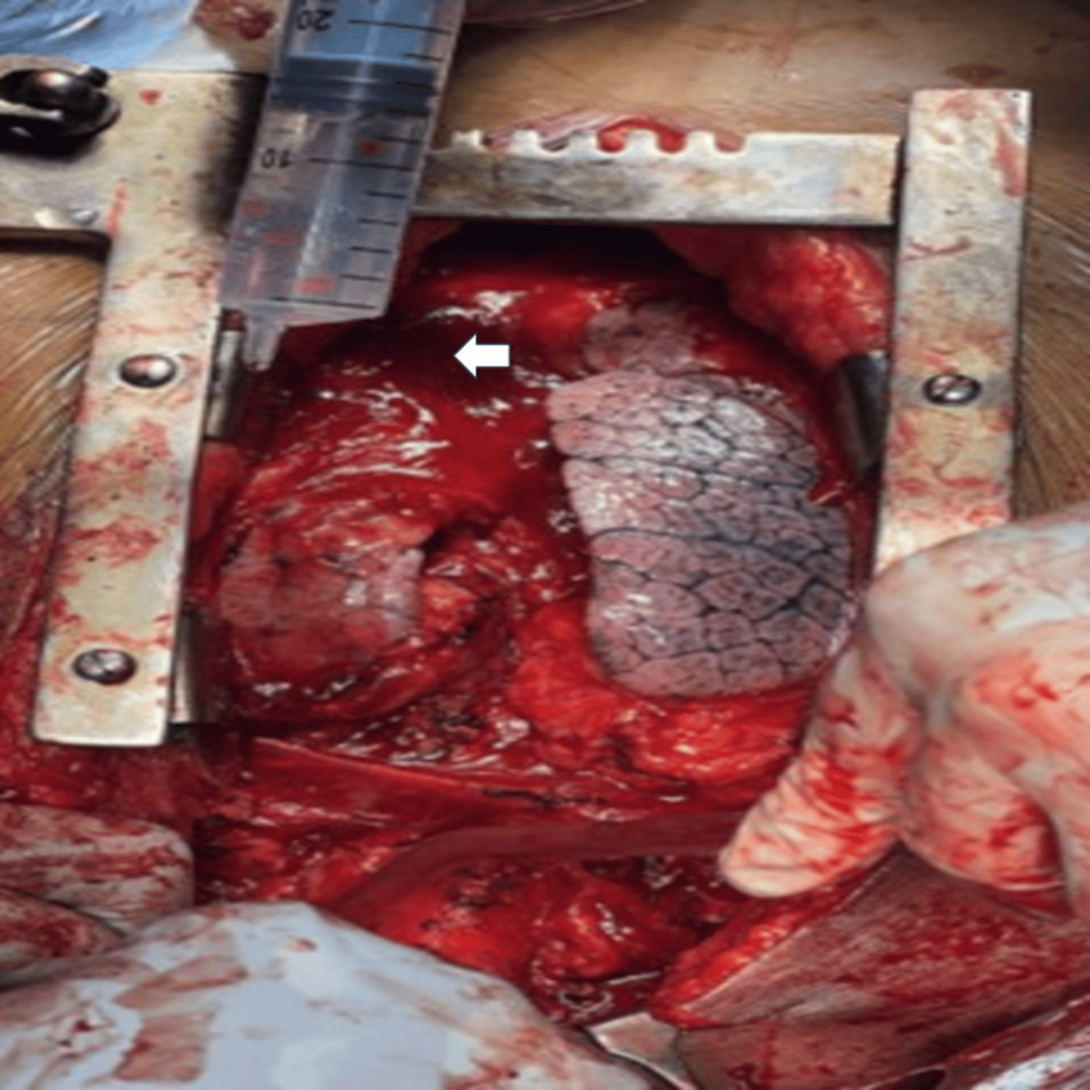
Intraoperative image showing retrosternal multinodular goiter (arrow) within the mediastinum The lobulated thyroid mass is seen in the operative field following sternotomy, with surrounding mediastinal soft tissues. Metallic retractors are visible on either side providing exposure, and surgical instruments are seen within the field. The superior aspect is oriented towards the top of the image and the inferior aspect towards the bottom.

The sternum was divided and retracted to expose the mediastinum. The retrosternal portion of the goiter was mobilized using gentle blunt and sharp dissection, carefully separating it from surrounding mediastinal structures, including the great vessels, without vascular injury. The mass was delivered into the cervical field, and total thyroidectomy was completed (Figure 2).

**Figure 3:**
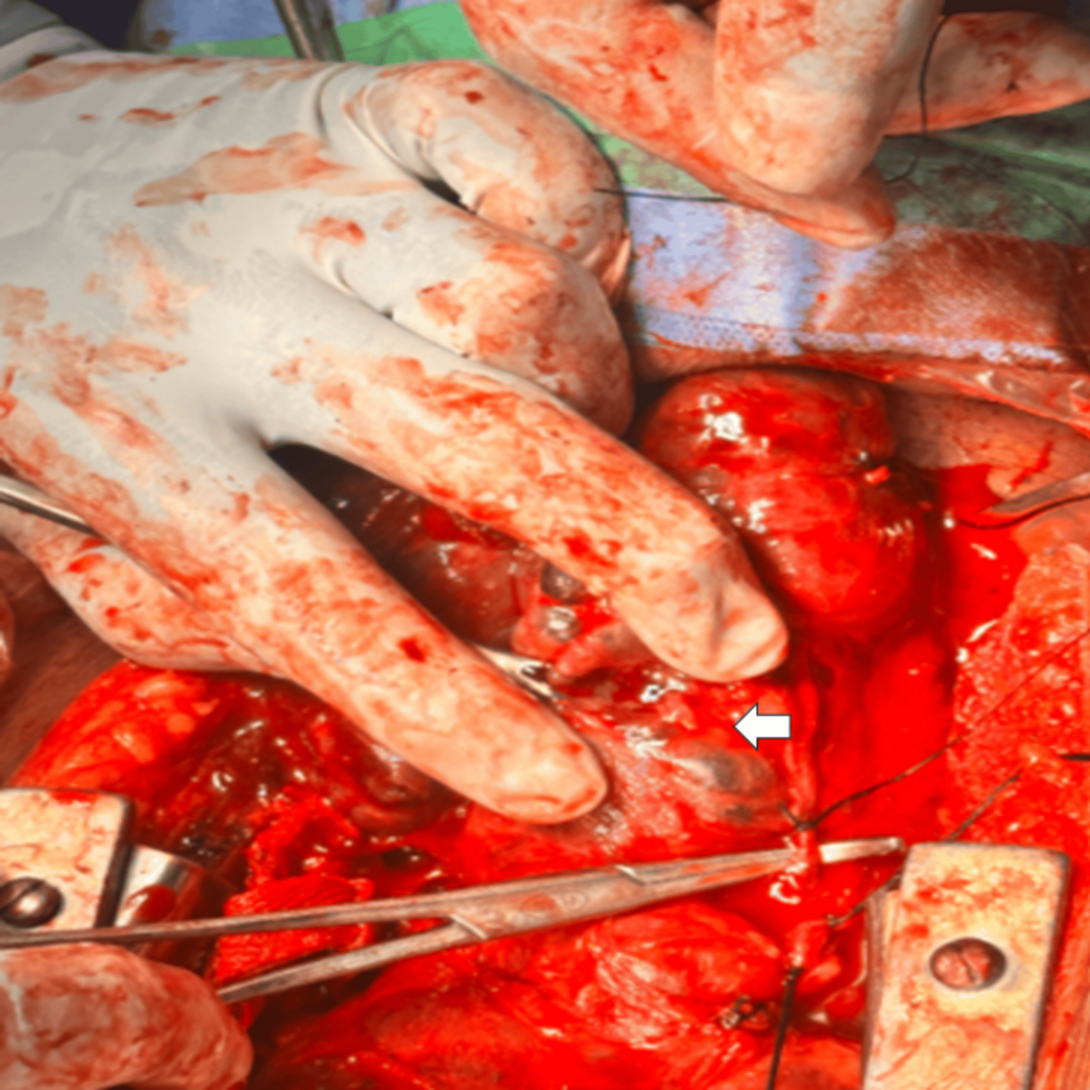
Intraoperative image showing retrosternal multinodular goiter (arrow) during sternotomy dissection The lesion is seen within the mediastinum, with surrounding soft tissues and surgical instruments. The sternum/retractor is seen, providing exposure of the operative field. The superior aspect is oriented towards the top of the image and the inferior aspect towards the bottom

**Figure 4:**
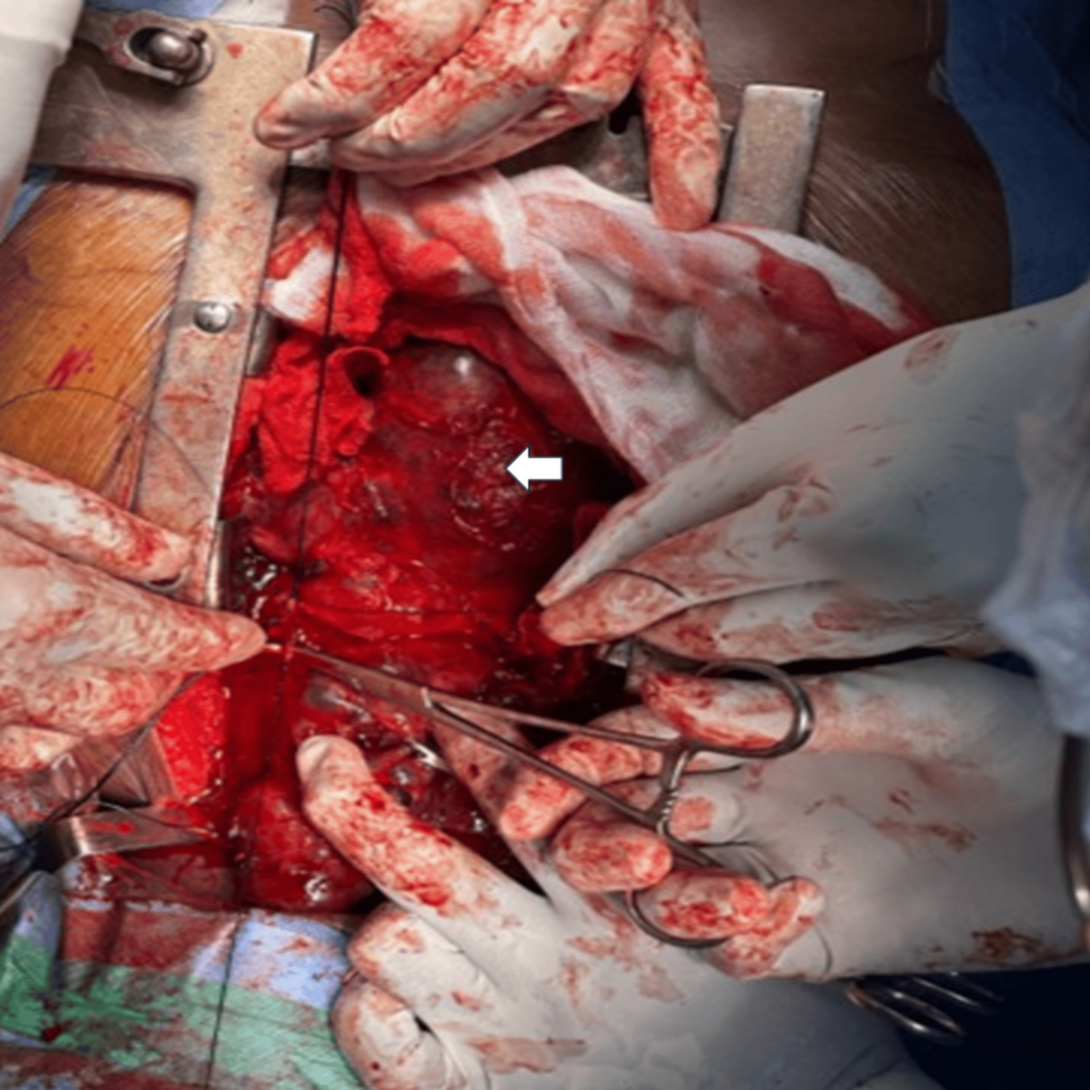
Intraoperative image showing mobilization of retrosternal multinodular goiter (arrow) from the mediastinum The lobulated thyroid mass is seen within the operative field, surrounded by mediastinal soft tissues. Surgical instruments including forceps and retractors are visible, facilitating dissection and delivery of the mass. The superior aspect is oriented towards the top of the image and the inferior aspect towards the bottom.

Intraoperatively, the mass was noted to extend into the superior and anterior mediastinum, with indentation over the right innominate artery without evidence of vascular encasement (Figures 3, 4).

Hemostasis was secured, and bilateral intercostal drains were placed in the mediastinum along with a suction drain in the neck. The sternum was closed with wires, followed by layered closure of the cervical incision.

Histopathological examination

**Figure 5:**
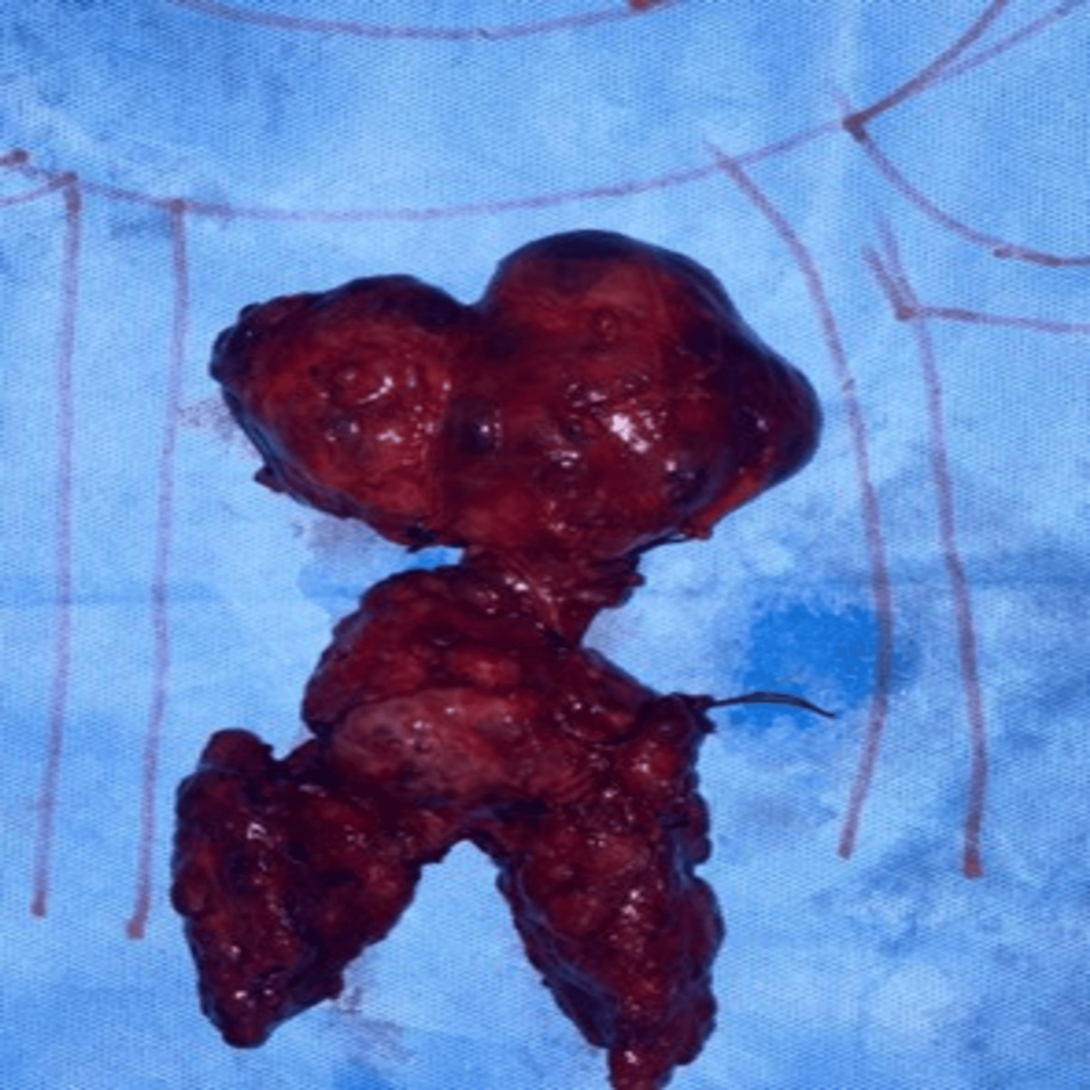
Gross specimen of excised multinodular thyroid gland with retrosternal mediastinal extension following combined cervical thyroidectomy and median sternotomy

The resected specimen weighed approximately 450 grams. The total thyroidectomy specimen measured 12 × 12 × 5 cm (Figure 5).

**Figure 6:**
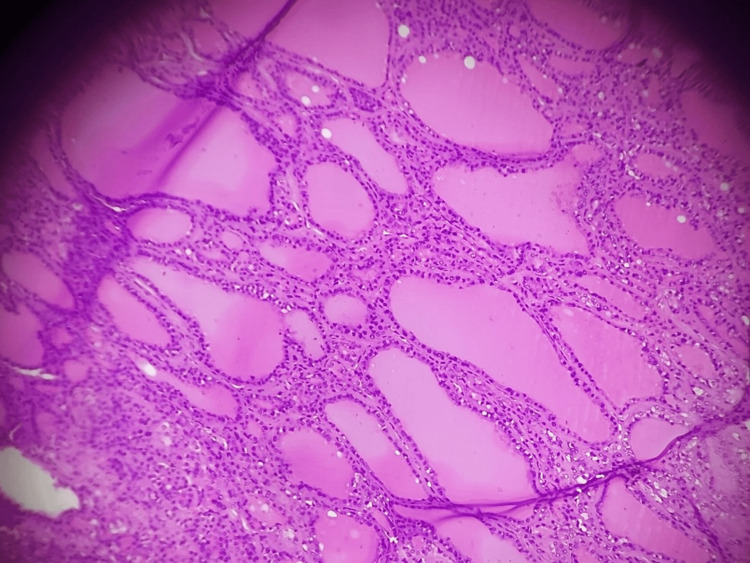
Histopathological image Hematoxylin and Eosin staining (H&E, ×100) showing variably sized thyroid follicles filled with abundant colloid and lined by flattened to cuboidal epithelium, consistent with multinodular goiter.

The retrosternal component measured approximately 10 × 5 × 7.5 × 4 cm. Histopathological examination confirmed multinodular goiter with retrosternal extension. Microscopy showed variably sized thyroid follicles filled with abundant colloid and lined by flattened to cuboidal epithelium, consistent with multinodular goiter. Adjacent involuted thymic tissue and small lymph nodes showed no specific pathology. There was no evidence of malignancy. Representative histopathological findings are shown in Figure 6.

Postoperative course

The postoperative period was uneventful. The patient remained hemodynamically stable with no evidence of airway compromise, hypocalcemia, or recurrent laryngeal nerve palsy. Drains were removed sequentially as output reduced. She was discharged on postoperative day nine in stable condition with advice for follow-up and thyroid hormone replacement therapy. The patient was followed up at two weeks and three months postoperatively. She remained asymptomatic with a well-healed surgical scar, normal calcium levels, and no clinical evidence of recurrent laryngeal nerve palsy. She continues on thyroid hormone replacement therapy and is under regular follow-up.

This case illustrates a large multinodular goiter with significant retrosternal extension requiring a combined cervicothoracic approach for safe and complete surgical excision.

## Discussion

Retrosternal goiter remains a surgically relevant entity because mediastinal extension directly influences operative exposure, airway management, and perioperative risk. CECT remains the cornerstone in assessing the extent, mediastinal relations, and planning the surgical approach in retrosternal goiter. The majority of cases represent secondary descent of a long-standing multinodular goiter and can often be managed through a cervical incision. However, a subset requires sternotomy depending on anatomical extent and mediastinal relations.

Ghabisha et al. reported 28 cases of retrosternal goiter managed successfully through a cervical approach without sternotomy, even in resource-limited settings [12]. In their series, careful blunt dissection and controlled traction allowed delivery of the intrathoracic component. In contrast, our case demonstrated inferior extension up to the level of D7 with close proximity to the aorta and indentation over the innominate artery. Given this depth of extension and mediastinal relation, a planned median sternotomy was performed to ensure vascular safety and complete excision. Unlike the cervical-only approach described by Ghabisha et al., our operative decision was driven by imaging-defined anatomical depth rather than resource limitation.

Cichoń et al. identified mediastinal depth and extension below the aortic arch as significant predictors for sternotomy [13]. In our patient, radiological assessment showed extension beyond the thoracic inlet with deep mediastinal involvement, consistent with the risk factors described in their study. This correlation supports the rationale for selecting a combined cervicosternotomy approach in our case.

Tikka et al. demonstrated that extension below the aortic arch and posterior mediastinal location independently predicted the need for an extracervical approach [14]. Our imaging findings revealed posterior abutment to the aorta and inferior extension to the D7 vertebral level. These features closely mirror the predictive factors identified by Tikka et al., further validating our decision for sternotomy.

Coskun et al. reported that sternotomy is required in a minority of cases, generally less than 10%, and is associated with longer operative time but acceptable morbidity [15]. In our patient, sternotomy was selectively employed due to extensive mediastinal spread. Despite the more invasive approach, the postoperative course was uneventful, with no airway compromise, vascular injury, or hypocalcemia, aligning with the acceptable morbidity profile described by Coskun et al.

Nankee et al. emphasized that most substernal goiters can be removed cervically and that sternotomy should be reserved for primary mediastinal goiter or deep posterior extension [16]. In our case, although the goiter was secondary in origin, the depth of mediastinal descent and proximity to major vascular structures warranted sternotomy. This highlights that while cervical removal remains standard, individualized operative planning based on radiological anatomy remains paramount.

Airway complications are a recognized concern in large retrosternal goiters. Shen et al. identified large gland size and tracheal compression as predictors of postoperative airway compromise [17]. Our patient had significant mediastinal extension but did not exhibit clinical tracheal deviation or respiratory distress preoperatively. Postoperatively, no airway complications occurred. This favorable outcome likely reflects preoperative imaging assessment and controlled mediastinal dissection under direct vision.

Simó et al. stressed that intrathoracic goiters should be managed in experienced centers with thoracic surgical collaboration when mediastinal extension is substantial [18]. In our case, cardiothoracic surgical assistance was obtained preoperatively. Intraoperatively, indentation over the right innominate artery was noted without encasement. The availability of thoracic expertise contributed to safe vascular dissection and complete resection, consistent with recommendations from Simó et al.

Rugiu et al. questioned the routine necessity of sternotomy and demonstrated that most retrosternal goiters can be delivered through a cervical incision [19]. However, they acknowledged that extension beyond the aortic arch or deep mediastinal positioning may necessitate extracervical access. Our case falls within this subset, as inferior extension reached up to the D7 level, supporting the need for sternotomy in selected patients.

Kumar et al., in an Indian cohort, reported that the majority of retrosternal goiters were multinodular and benign, with sternotomy required in a small proportion of cases [20]. Our histopathology similarly confirmed multinodular goiter without malignancy. The benign nature of the pathology aligns with the clinical profile described in the Indian population, reinforcing that mediastinal extension does not necessarily imply malignancy but increases technical complexity.

Berri et al. reported increased complication rates in large goiters, including hypocalcemia and recurrent laryngeal nerve injury [21]. In our patient, total thyroidectomy with sternotomy was performed without postoperative hypocalcemia, nerve palsy, or major complications. The uneventful recovery underscores the importance of meticulous surgical technique, identification of recurrent laryngeal nerves, preservation of parathyroid glands, and structured postoperative monitoring. Intraoperative nerve monitoring was not utilized in this case. However, meticulous surgical dissection with direct visual identification and preservation of the recurrent laryngeal nerves was performed. There was no postoperative evidence of recurrent laryngeal nerve palsy.

Overall, our case demonstrates that while most retrosternal goiters may be managed through a cervical incision, extensive inferior mediastinal extension and proximity to major vascular structures justify a planned sternotomy. The findings correlate closely with predictive factors described in the literature. Careful radiological evaluation, multidisciplinary coordination, and individualized surgical strategy remain the cornerstone of safe management in retrosternal goiter.

## Conclusions

Retrosternal goiter represents a technically demanding variant of multinodular thyroid enlargement due to mediastinal extension and proximity to major vascular structures. Although most cases can be managed through a cervical approach, extensive inferior descent and close mediastinal relations warrant a planned sternotomy to ensure safe dissection and complete excision.

In the present case, preoperative contrast-enhanced imaging accurately defined the depth of mediastinal extension and guided operative planning. Multidisciplinary coordination with cardiothoracic surgery enabled controlled mediastinal access and prevention of vascular injury. Despite significant extension, the patient had an uneventful postoperative course without airway compromise, recurrent laryngeal nerve palsy, or hypocalcemia.

This case underscores the importance of individualized surgical strategy based on radiological anatomy rather than gland size alone. Careful preoperative assessment, structured intraoperative planning, and meticulous technique remain fundamental to achieving favorable outcomes in retrosternal goiter surgery.
